# Benzo*[a]*pyrene, Aflatoxine B_1_ and Acetaldehyde Mutational Patterns in *TP53* Gene Using a Functional Assay: Relevance to Human Cancer Aetiology

**DOI:** 10.1371/journal.pone.0030921

**Published:** 2012-02-03

**Authors:** Vincent Paget, Mathilde Lechevrel, Véronique André, Jérémie Le Goff, Didier Pottier, Sylvain Billet, Guillaume Garçon, Pirouz Shirali, François Sichel

**Affiliations:** 1 Groupe Régional d'Etudes sur le Cancer-EA 1772, Université de Caen Basse-Normandie, Caen, France; 2 Groupe Régional d'Etudes sur le Cancer, Centre Régional de Lutte Contre le Cancer François Baclesse, Caen, France; 3 Unité de Chimie Environnementale et Interactions sur le Vivant-EA 4492, Université du Littoral Côte d'Opale, Dunkerque, France; 4 Université Lille Nord de France, Lille, France; Shantou University Medical College, China

## Abstract

Mutations in the *TP53* gene are the most common alterations in human tumours. *TP53* mutational patterns have sometimes been linked to carcinogen exposure. In hepatocellular carcinoma, a specific G>T transversion on codon 249 is classically described as a fingerprint of aflatoxin B_1_ exposure. Likewise G>T transversions in codons 157 and 158 have been related to tobacco exposure in human lung cancers. However, controversies remain about the interpretation of *TP53* mutational pattern in tumours as the fingerprint of genotoxin exposure. By using a functional assay, the Functional Analysis of Separated Alleles in Yeast (FASAY), the present study depicts the mutational pattern of *TP53* in normal human fibroblasts after *in vitro* exposure to well-known carcinogens: benzo*[a]*pyrene, aflatoxin B_1_ and acetaldehyde. These *in vitro* patterns of mutations were then compared to those found in human tumours by using the IARC database of *TP53* mutations. The results show that the *TP53* mutational patterns found in human tumours can be only partly ascribed to genotoxin exposure. A complex interplay between the functional impact of the mutations on p53 phenotype and the cancer natural history may affect these patterns. However, our results strongly support that genotoxins exposure plays a major role in the aetiology of the considered cancers.

## Introduction

Somatic mutations are a key event in carcinogenesis and it is now well recognised that carcinogenesis involves heritable genetic factors as well as epigenetic changes. The turning point during the cancer initiation is the acquisition of targeted somatic mutations which confers to cells a selective advantage for clonal proliferation. Among the many different genes involved in this process, *TP53* remains the most frequently mutated in human cancers, accounting for over 70% of all the mutations described so far in human cancers [1]. The International Agency for Research on Cancer (IARC) *TP53* mutation database contains 27,580 somatic *TP53* mutations (http://www-p53.iarc.fr/, R15 release) [2]. In contrast to other tumour suppressor genes, the vast majority of *TP53* mutations are missense rather than non sense or frameshift mutations, leading to various patterns in different cancers. Interestingly, these patterns have been sometimes linked to exposure of aetiological factors such as UV light in skin carcinoma [3], tobacco in lung cancers [4], aflatoxin B_1_ in hepatocarcinoma [5] or aristolochic acid in renal carcinoma [6], [7]. However, interpretation of *TP53* mutational pattern in tumours as a fingerprint of genotoxin exposure remains controversial [8]. Indeed, mutations resulting from exposure to genotoxins and other cellular events arising during carcinogenesis affect the final mutational profile found in human tumours, e.g. clonal selection of particular mutants, the genetic instability in cancerous cells or epigenetic influences (e.g. methylation status) [9]–[11].

As many *TP53* mutations found in tumours strongly affect p53 functional properties [12], a functional assay, the Functional Analysis of Separated Alleles in Yeast (FASAY) appears as an useful technique to distinguish between silent mutations in *TP53* and those that render the protein inactive [13], [14]. DNA from a variety of sources can be screened by FASAY for the presence of a *TP53* allele encoding a dysfunctional protein. We previously described mutational patterns in normal human fibroblasts after *in vitro* exposure to acetaldehyde and aflatoxin B_1_ (AFB_1_) by using FASAY [15], [16]. Here, we have completed these data with those obtained with a well-known genotoxin: benzo*[a]*pyrene (B*[a]*P). Exposure to B*[a]*P and AFB_1_ results in the formation of bulky adducts predominantly targeting guanine. We will discuss how these may lead to functionally different mutational patterns in different tumours.

Further, the experimentally-generated *TP53* mutational patterns were compared with those in related tumours recorded in the IARC database [2]. The mutational pattern of B*[a]*P was compared to those of lung cancers and discussed thoroughly in the present paper. This enabled us to discuss the causality of genotoxins exposure to the human cancer mutational patterns.

## Materials and Methods

### Cell line and cell culture

Human diploid fibroblasts AG1521 (Coriell Institute, Camden, NJ) were chosen for this study. Absence of pre-existing mutations in *TP53* locus in these cells was confirmed by sequencing genomic DNA with a Beckman Automated Sequencer (CEQ 8000). Cells were cultured at 37°C in a humidified atmosphere of 5% CO_2_ and 95% air, in Eagle's Minimal Essential Medium (MEM) supplemented with 10% inactivated foetal bovine serum (FBS). Before exposure to the genotoxin, the medium was completed with 200 µl (4%) of S9 liver microsomal fraction of phenobarbital- and naphtoflavone-treated rats (Biopredic, Rennes, France), NADPH (4 µM) and G6P (5 mM). The required amount of B*[a]*P (Sigma, CAS number: 50-32-8, up to 200 µM) was diluted in the above medium and used to treat cells for 2 h at 37°C. Post-exposure, the culture plates were washed three times with PBS and incubated in fresh medium. As the doubling time of these cells is around 3 days, incubation period of one week was chosen to allow two cell cycles and ensure fixation of mutations.

### Cytotoxicity study

AG1521 fibroblasts were exposed to B*[a]*P at concentrations from 1 to 200 µM in the presence of S9, for 2 h at 37°C in 96-well micro-plates. The plates were washed three times with PBS and incubated for 3 days at 37°C. Cytotoxicity was evaluated with an XTT assay (Sigma) according to the Scudiero protocol [17].

### Post-labelling

Post-labelling method was adapted from Reddy and Randerath [18]. Genomic DNA was extracted using the classical phenol/chloroform method after treatment by RNAse A and T1 followed by proteinase K (Sigma-Aldrich) and post-labelling assay was then performed as previously described [19]. From the autoradiograms thus obtained, spots corresponding to the adducts were excised for measuring radioactivity. Results are expressed as Relative Adduct Levels (RAL) calculated as followed: RAL = (cpm_adducts_/cpm_BPDE_)×110.7×10^−8^, where cpm_adducts_ and cpm_BPDE_ correspond to counts per minute from the excised spots and from the positive control issued from calf thymus exposed to B*[a]*P-7,8-Dihydrodiol-9,10-Epoxide (BPDE), and carrying 110.7 adducts for 10^−8^ normal nucleotides as measured elsewhere [20]. Positive control was kindly supplied by F. Beland, Jefferson, USA.

### FASAY

RNA extraction and Reverse Transcription (RT)-PCR were performed as previously described [15]. To amplify the cDNA primers containing a phosphorothioate linkage at the 3′ end were designed : P3 [5′-ATT-TGA-TGC-TGT-CCC-CGG-ACG-ATA-TTG-AA(s)C-3′] and P4 [5′-ACC-CTT-TTT-GGA-CTT-CAG-GTG-GCT-GGA-GT(s)G-3′]. FASAY was done according to Flaman *et al.* [14], with some modifications as previously described [15]. Plasmid pSS16 and yeast strain YIG397 were generously donated by J.M. Flaman (INSERM U614, Rouen, France) and J. Cachot (LEMA EA3222, Le Havre, France), respectively.

*TP53*-expressing plasmids were rescued from isolated red colonies which were returned to culture and lysed with *Zymolyase* (MP-Biomedicals). Purelink Quick Plasmid Miniprep Kit® (Invitrogen) was used to rescue the plasmids. Before sequencing, *TP53* cDNA was re-amplified with primers P5 [5′-TCT-GTC-ACT-TGC-ACG-TAC-TCC-3′] and P6 [5′-AGA-GGA-GCT-GGT-GTT-GTT-GG-3′], designed to cover exon 4–9 according to sequence described in GenBank (NM_000546). Two sets of primers were chosen to sequence the recombination sites on either side of the *TP53* cDNA: at 5′ region primers PA [5′-CAG-TCA-GAT-CCT-AGC-GTC-GAG-3′] and PB [5′-CTC-CGT-CAT-GTG-CTG-TGA-CT-3′]; at 3′ region primers PC [5′-AAG-GAA-ATT-TGC-GTG-TGG-AG-3′] and PD [5′-CAG-GCC-CTT-CTG-TCT-TGAAC-3′].

### Sequence tools analysis

The Thierry Soussi database (http://p53.free.fr/) was used a reference for the *TP53* wild type sequence and the IARC database R14 release (http://www-p53.iarc.fr/) was used for comparing different *TP53* mutational patterns [2]. BLAST tool from NCBI (http://www.ncbi.nlm.nih.gov/) was used for analysis of sequence similarities.

### Statistical analysis

Statistical analysis was done using the SAS Software release 9.2. A chi square test with one degree of freedom was applied to evaluate the correspondence between the observed and theoretical frequencies of mutations out of the base substitutions in exons 4 to 9. Comparisons between the substitutions patterns pertaining to the genotoxins were done using a Fisher's exact test. All tests were two sided with a significance level of 0.05.

## Results

### Cytotoxicity studies on AG1521 fibroblasts

**Figure 1:**
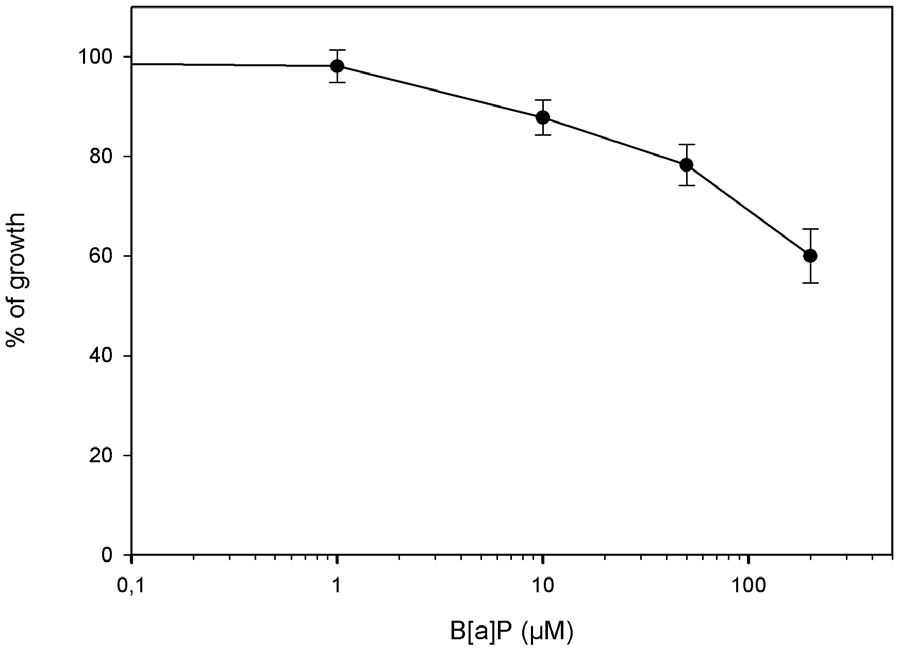
Cytotoxicity of B*[a]*P. Cytotoxicity of B*[a]*P to human fibroblastsAG1521 after 2 h exposure and 3 days recovery (4 wells per concentration, means and standard deviations).

Cell growth after 2 hours exposure of AG1521 human fibroblasts to B*[a]*P at various concentrations showed a moderate cytotoxicity of B*[a]*P in these conditions, the IC50 was estimated to be above 200 µM (Figure 1). We then chose exposure concentrations ranging from 1 µM corresponding to the higher concentration without any growth inhibition to 50 µM corresponding to a 25% growth inhibition. Thus, cellular damage was minimized and resumption of culture was easier after exposure. The range of concentrations of acetaldehyde and AFB_1_ used were previously described and discussed [15], [16]. Acetaldehyde was used at 0.1–7.5 mM and AFB_1_ at 16–800 nM.

### Post labelling of B*[a]*P induced DNA adducts

**Figure 2:**
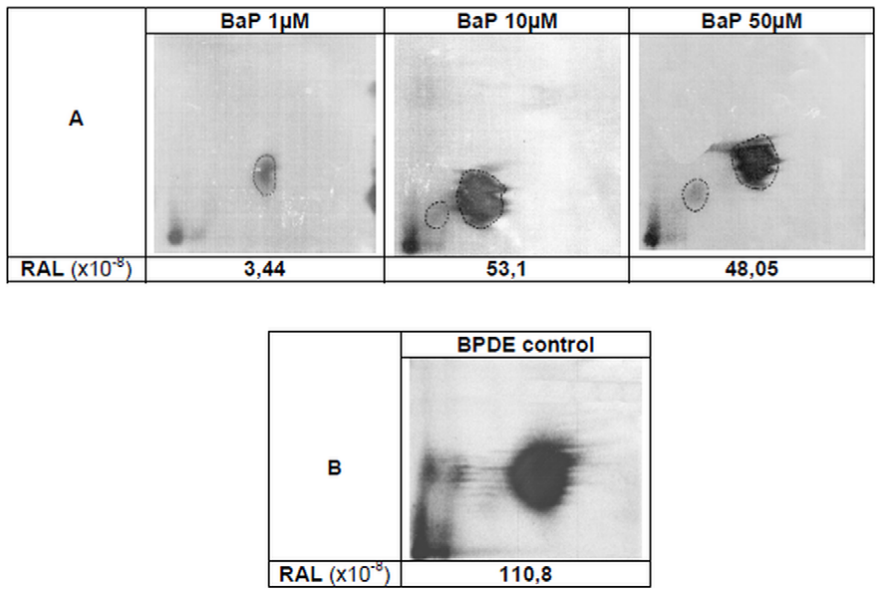
Post-labelling DNA adduct patterns. (**A**) AG1521 cells after 2 hours exposure to B*[a]*P. (**B**) Calf thymus DNA after exposure to 35 nM B(a)P-7,8-Dihydrodiol-9,10-Epoxide (BPDE control). RAL values are the mean of two independent assays.

Figure 2A shows the pattern of DNA bulky adducts after 2 hours exposure to B*[a]*P 1, 10 and 50 µM in the presence of exogenous metabolization fraction (S9mix). Positive control was obtained from naked calf thymus DNA exposed to 35 nM BPDE, the active metabolite (Figure 2B). A major spot migrating similarly on the thin layer chromatography plates was visible in the four samples. After B*[a]*P exposure, a second but very faint spot was also observed for the 2 highest concentrations. The RAL in DNA from B*[a]*P-exposed cells after exogenous metabolization were significantly above the limit of quantification of 1.75×10^−8^. This value was determined after an internal process of validation (unpublished data). This demonstrates the efficiency of the metabolizing procedure. However, a plateau effect is reached at 10 µM.

### Mutation rates in FASAY

Table 1 shows the overall results for the B*[a]*P. Each concentration was tested in triplicate. As reproducibility among triplicates was very high, the total number of colonies for each concentration is shown.

**Table 1:** Summary of yeast transformations for B*[a]*P experiments.

			Benzo*[a]*pyrene (µM)		
		Control cells	1	10	50
***Yeast transformation***					
Total number of colonies	A	4084	4890	5423	5240
Total number of red colonies	B	97	148	220	181
***Recovering of red colonies and PCR step***					
Analysed red colonies	C	48	40	84	66
Red colonies bearing *TP53* (PCR positive)	D	37	39	77	60
Red colonies bearing non digested pSS16 (C–D)	E	11	1	7	6
***Sequencing step***					
Colonies mutated on exons 4 to 9	F	3	8	22	15
AGT insertion in exon 7/8 (splicing defect)	G	2	2	3	3
“True” mutations (F–G)	H	1	6	19	12
***Rate of mutation***					
Total number of “true” mutations (HxB/C)		2.0^a^	22.2^a^	49.8^a^	32.9^a^
Rate of mutation (%) 100×(HxB/C)/A		**0.05**	**0.45**	**0.92**	**0.63**

Data pooled from triplicates are shown.

a Estimated values.

Among the red colonies arising from untreated control cells, only a few bear mutations in the central part of *TP53*, i.e. exons 4 to 9. The other red colonies were considered as artefacts as they carried undigested pSS16 plasmid or mutations located in the recombination sites. Among the 3 red colonies from control cells bearing a mutation in the central part of *TP53*, 2 showed an AGT insertion in exon 7/8 which is likely a splicing defect rather than a true mutational event, as described earlier [15]. It is noteworthy that the rate of this splicing defect was comparable among the experiments on the three genotoxins discussed here. Only one true mutation was found in the control cells, allowing the calculation of a spontaneous mutation rate of 0.05%. This rate was around 9 to 18 times lower than the mutation rates calculated in exposed cells. This single spontaneous mutation, was a G>A transition within a CpG sequence in codon 267 (CGG>TGG). This kind of mutation is classically ascribed to spontaneous deamination of 5-methylcytosine, although we cannot exclude the possibility of an error introduced by the DNA polymerase during the RT-PCR step.

The mutation rates in exposed cells were comprised between 0.45 to 0.92%. As observed with post-labelling data, a plateau is reached at 10 µM.

### Identification of *TP53* mutations in B*[a]*P exposed AG1521 human fibroblasts

**Figure 3:**
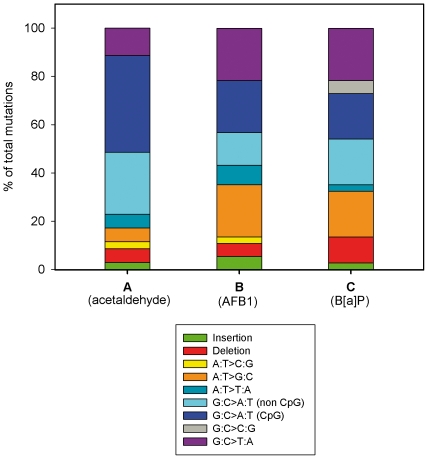
*In vitro* induced mutation patterns of *TP53*. (**A**) *In vitro* Acetaldehyde-exposed human fibroblasts AG1521 (FASAY) (n = 35 mutations, data from [15]), (**B**) *In vitro* AFB_1_-exposed human fibroblasts AG1521 (FASAY) (n = 37 mutations, data from [16]), (**C**) *In vitro* B*[a]*P-exposed human fibroblasts AG1521 (FASAY) (n = 37 mutations).

Table 2 shows the 37 true mutations found in B*[a]*P-exposed cells. Five mutations were one- or more-base insertions/deletions, leading to a frameshift. The remaining mutations were single nucleotide substitutions including 1 nonsense and 25 missense mutations. The mutational pattern is depicted in Figure 3C: G>T transversions were the most prevalent (8/37, 21.6%). They are followed by G>A, G>A at CpG and A>G transitions (7/37, 18.9% for each one of them), deletions (4/37, 10.8%), G>C transversions (2/37, 5.4%), A>T transversions (1/37, 2.7%) and one base insertion (1/37, 2.7%). This mutational pattern is then discussed in comparison with that previously obtained after AG1521 fibroblasts exposure to acetaldehyde (Figure 3A) and aflatoxin B_1_ (AFB_1_, Figure 3B) [15], [16].

**Table 2:** Summary of *TP53* mutations found in B*[a]*P exposed-cells (Cc: concentration).

Number	Cc (µM)	Exon	Codon	Base change	Amino acid substitution	Nature of mutation
1	50	4	104	CA**G**→CA**T**	Gln→His	Missense
2	50	4	117	G**G**G→G**T**G	Gly→Val	Missense
3	1	5	126	T**A**C→T**T**C	Tyr→Phe	Missense
4	1	5	127	T**CC** →T**T**C	Ser→Phe	Missense
5	10	5	127	**T**CC→ **C**CC	Ser→Pro	Missense
6	10	5	127	T**C**C→T**G**C	Ser→Cys	Missense
7	10	5	128	C**C**T→C**T**T	Pro→Leu	Missense
8	10	5	138	GCC AAG→G**T**C CAA G		Frameshift
9	50	5	141	**T**GC→ **C**GC	Cys→Arg	Missense
10	50	5	152	C**C**G→C**TG** (CpG)	Pro→Leu	Missense
11	10	5	163	TA**C** →TA**A**	Tyr→Stop	Nonsense
12	10	5	175	C**G**C→C**A**C (CpG)	Arg→His	Missense
13	10	5	177	C**C**C→C**A**C	Pro→His	Missense
14	10	5	179	**C**AT→ **T**AT	His→Tyr	Missense
15	1	5	181	**C**GC→ **A**GC	Arg→Ser	Missense
16	50	6	224	**G**AG→ **A**AG	Glu→Lys	Missense
17–18	10,50	7	234	**T**AC→ **C**AC	Tyr→His	Missense
19	50	7	236	8 bases deletion		Frameshift
20	1	7	242	T**GC** →T**A**C	Cys→Tyr	Missense
21	10	7	248	C**G**G→C**C**G	Arg→Pro	Missense
22–23–24	10,10,50	7	248	C**G**G→C**A**G (CpG)	Arg→Gln	Missense
25	1	7	251	A**T**C→A**C**C	Ile→Thr	Missense
26	10	7	260	23 bases deletion		Frameshift
27	50	7	260	24 bases deletion		Frameshift
28	10	8	271	GA**G** →GA**T**	Glu→Asp	Missense
29	10	8	277	T**G**T→T**A**T	Cys→Tyr	Missense
30	50	8	277	T**G**T→T**T**T	Cys→Phe	Missense
31	50	8	279	G**G**G→G**A**G	Gly→Glu	Missense
32	10	8	280	**A**GA→ **G**GA	Arg→Gly	Missense
33–34	10,10	8	283	C**G**C→C**A**C (CpG)	Arg→His	Missense
35	10	8	285	GA**G** →GA**T**	Glu→Asp	Missense
36	10	8	288	**A**AT→ **G**AT	Asn→Asp	Missense
37	1	8	290	1 base deletion		Frameshift

### Localization of mutations among *TP53* exons 4 to 9

**Figure 4:**
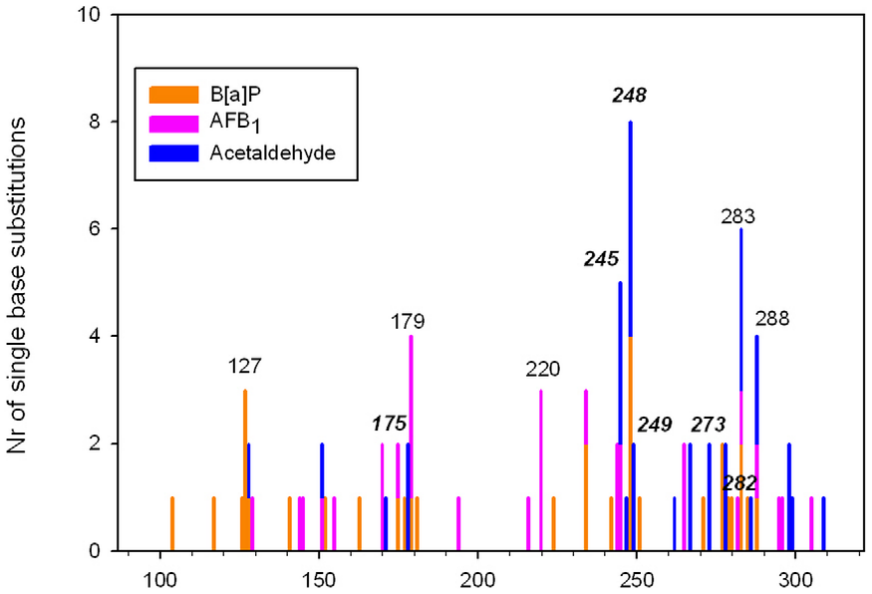
Codon distribution of single base substitutions in the *TP53* gene. Mutational pattern as seen in AG1521 fibroblasts after B*[a]*P (orange bars, n = 32 mutations); AFB_1_ (pink bars, n = 29 mutations); and acetaldehyde (blue bars, n = 32 mutations) exposure. Codons typed in italic are major hot-spots in human tumours according to IARC database.

Figure 4 shows the localization of mutations from B*[a]*P (this study), AFB_1_ and acetaldehyde [15], [16]. B*[a]*P pattern shows two hot-spots, codons 248 and 127, whereas AFB_1_ hot-spots are codons 179 and 220 and acetaldehyde ones codons 248, 245 and 283.

In order to assess if the pattern of B*[a]*P mutations could be a matter of chance, we analysed the data as described by Shen *et al.* [21]. For this analysis of hot-spots, only base substitutions occurring in exons 5 to 9 were taken into account. Hot-spots were defined as the 24 mutations that account for 50% of the mutations reported in lung cancer in the R14 IARC database. In order of decreasing frequency, these are codons: 273, 248, 249, 245, 158, 157, 175, 179, 282, 220, 242, 163, 176, 154, 280, 266, 298, 237, 193, 281, 159, 135, 234 and 244. If these 24 hot-spots were assumed to be randomly mutated, then they would be predicted to be hit 24/205 (total number of codons in exons 5 to 9), i.e. at a frequency of 11.7%. Out of the 30 base substitutions in exons 5 to 9 in our study, 11 (36.7%) correspond to one of the above hotspots identified for lung cancers, a rate significantly higher than that predicted for random events (11.7%; p = 2.10^−5^). The same analysis was performed using either only hot-spots found in lung cancer from smokers, or another definition of hot-spots, i.e. mutation frequency superior to 2% of all mutations as proposed by Olivier *et al.* [22]. These analyses were highly significant whatever the parameters used (data not shown).

These observations, together with the comparison of mutation sites with the lung cancer hot-spots as shown above, strongly argue against a random distribution of mutations in our assay.

## Discussion

We will address two important issues: first, are experimentally generated mutation profiles representative of those occurring naturally for a given genotoxin? Secondly, based on a compilation of experimental and epidemiological data on mutations associated with human tumours, can chemically-induced mutations of *TP53* at specific sites (codons) be identified as a key event in carcinogenesis?

### Relationship between B*[a]*P mutagenicity and *TP53* mutations

Many genotoxic metabolites are produced from B*[a]*P. The main way, which involves CYP1A, CYP1B, CYP3A and epoxide hydrolases, leads to the ultimate genotoxic metabolite (±)-anti-BPDE which binds to DNA and forms predominantly DNA adducts at the *N2* position of guanine and to a lesser extent at the *N6* position of adenine [23]–[25]. Another pathway involves dihydrodiol dehydrogenases AKR1A1 and 1C1-4 which oxidize B*[a]*P dihydrodiols to catechols [26]. Catechols can then undergo mono-electronic oxidizations leading to *o*-quinones which form DNA adducts, such as the B*[a]*P-7,8-dione-*N^2^*-dG and the B*[a]*P-7,8-dione-*N^6^*-dA [27], [28]. In this study, during the exposure of AG1521 fibroblasts to B*[a]*P, metabolizing enzymes were supplied exogenously, according to the standard practices in genotoxicity testing. The post-labelling analysis revealed a major spot that co-migrated with that seen in control DNA exposed to BPDE. This strongly suggests that B*[a]*P exposure results predominantly in BPDE-DNA adducts.

In agreement with the data in literature, we found that the majority of base substitutions induced by B*[a]*P were located on G:C base pairs of which only 6 within CpG regions. Guanines in methylated CpG sequences are typical targets for DNA adducts [10], [23]. It is likely that DNA methylation is poor in a cellular model such as the one employed here, but Shen *et al.*, using the same reporter yeast strain transformed by chemically modified plasmids, observed that DNA methylation did not affect the generation of BPDE adducts [21].

Using UvrABC endonuclease in combination with ligation-mediated PCR (LMPCR), the distribution of BPDE adducts on exons 5, 7 and 8 of *TP53* in HeLa cells have been mapped [29], [30]. Strong adduct formation occurred at the guanines in codons 154, 156, 157, 158, 159, 245, 248 and 273, most of which are described as hot-spots in lung cancers (157, 158, 245, 248, and 273; IARC database, version R14). Indeed, the codon 248 was most frequently targeted by B*[a]*P in our assay. Secondly, using methylated and BPDE-exposed DNA fragments containing exon 5 of *TP53*, adducts were found at three other codons: 152, 175, 181, all of which were uncovered in the present study as well [10].

B*[a]*P induced mutational patterns have been extensively studied in many genes in various biological systems. Assays conducted in rodent cells *in vitro* or *in vivo* on reporter genes such as *HPRT* or *LacI* have consistently reported the predominance of G>T transversions and, to a lesser extent, of G>A transitions and deletions [31]–[34]. The rate of G>T transversions seems to be concentration-dependant [31]. Interestingly, using a yeast assay with various strains deficient in nucleotide excision repair, it was demonstrated that DNA polymerases η and ζ were required in combination for the translesion repair across BPDE adducts, leading to G>T transversions [35]. Pol ζ alone was also able to generate large deletions in this assay.

**Figure 5:**
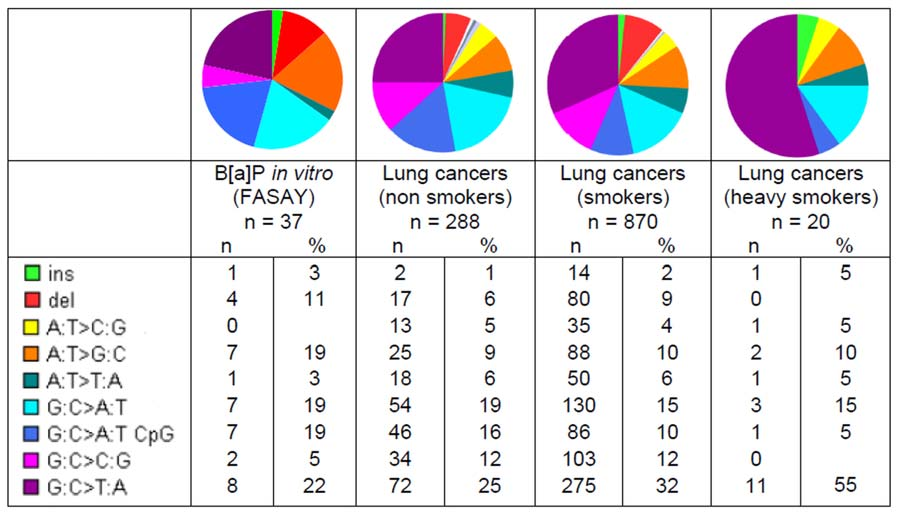
Mutation patterns of *TP53*: comparison *vitro-vivo*. From left to right: *in vitro* B*[a]*P-exposed human fibroblasts AG1521 (FASAY) (n = 37); human lung cancers, non smokers (n = 288); human lung cancers, smokers (n = 870); human lung cancers, heavy smokers (n = 20). Lung cancer data from IARC database R14. Rare mutations were omitted in these patterns.

Depending on the experimental approach, discrepancies have been observed between mutational patterns in *TP53* induced by *in vitro* and *in vivo* exposure to B*[a]*P or BPDE. Using the same yeast assay as us, but after a direct BPDE exposure of a methylated plasmid bearing the human *TP53* sequence, Yoon *et al.* have found a clear predominance of G>T transversions whereas Shen *et al.* have found mainly G>A transitions [11], [21]. The direct sequencing of *TP53* in human-p53 knock-in (Hupki) murine embryonic fibroblasts after *in vitro* exposure to B*[a]*P have shown mainly G>T transversions [36], [37]. Similarly, G>T mutations were also found predominantly in skin tumours after B*[a]*P exposure [38]. The sequencing of *TP53* from various tumours induced after *in vivo* exposure of mice to B*[a]*P or coal tar led to the same extent of G>T, G>A and G>C mutations [39]. On the whole, it is clear from the vast majority of studies, that regardless of the gene being studied and the biological system used, G>T transversion is the hallmark of B*[a]*P or BPDE exposure. In this regard, we were surprised to find these transversions accounting for only 22% of mutations in our study, but rising from 17% at 1 µM to 27% at 50 µM. As suggested by Wei *et al.* [31], it may be a matter of dose. Many *in vitro* studies have used BPDE in the µM range [11], [35] whereas we used B*[a]*P in the same range, which obviously led to a lower exposure to BPDE in our protocol. Some *in vitro* studies used B*[a]*P in the µM range, but in these studies the time of exposure ranged from 4 to 9 days instead of the 2 hours in our protocol [36], [37]. This suggests that the G>T transversions could be the hallmark of a strong exposure to B*[a]*P, a scenario which appears plausible in the context of tobacco smoke exposure in heavy smokers, whereas moderate exposure could lead to both G>T, G>A, G>C and deletions, as found in many studies. This hypothesis is supported by the prevalence of G>T transversions in lung cancers with respect to tobacco exposure. According to IARC database (Figure 5), this prevalence ranges from 25% in non smokers to 32% in smokers and 55% in heavy smokers.

Conversely to the rate of G>T transversion, we found a high rate of G>A transitions (38%), half of which being located in CpG sequences. This could be partly explained by spontaneous mutations as seen in control cells. But a technical bias may be considered since our protocol did not distinguish between two independently arising but identical mutation events and one of the following situation: (i) multiple mRNA transcripts from the same mutant cell that are subsequently independently captured into plasmids or (ii) clonal expansion of a mutant cell before harvesting of the pool of treated cells. In Table 2 only 3 mutations may be potentially relevant of such bias, suggesting that artefactual duplication of mutations might be infrequent. In addition, duplicated mutations are mostly G>A transitions in CpG sites. This may wrongly increase the rate of this particular mutation.

### Comparison of *TP53* mutational profiles obtained for the genotoxins, B*[a]*P, acetaldehyde and AFB_1_

Results obtained from the application of FASAY to evaluate mutagenic effects of *in vitro* exposure of normal human fibroblasts to B*[a]*P, acetaldehyde and AFB_1_ were compared. Fisher's exact test revealed that in terms of the type of mutation, B*[a]*P and AFB_1_ patterns were not statistically different (p = 0.6735), whereas the differences between the patterns for acetaldehyde and either B*[a]*P or AFB_1_ were statistically of borderline significance (p values of 0.0546 and 0.0705, respectively). These three compounds are known to cause various types of DNA damages which could in turn, be related to specific mutations. Acetaldehyde-induced pattern is mainly characterized by a very high proportion of G>A transitions (66%), mostly located within CpG sequences which is a likely basis for the formation of interstrand crosslink adducts in CpG sequences [15]. In contrast, the high rate of G>T transversions observed among B*[a]*P- and AFB_1_-induced mutations, has been recognized as a hallmark of their mutagenicity, *in vitro* and *in vivo* [4], [40]. This is mostly attributable to the presence of bulky adducts.

B*[a]*P pattern reveals two unique features in comparison to those of AFB_1_ and acetaldehyde: the occurrence of G>C transitions and a higher rate of deletions (11% vs 5–6%). Whereas AFB_1_ and acetaldehyde induced single-base deletions, B*[a]*P could generate deletions up to 24 nucleotides. This feature may be the result of a particular translesion synthesis pathway of B*[a]*P adducts as discussed above.

Thus, a preliminary analysis using the FASAY enabled us to record and distinguish different experimentally-generated mutation patterns in *TP53*, which were then used to compare with existing epidemiological data on maps of DNA adducts and tumour mutations in *TP53*.

### Occurrence of experimental *TP53* mutants in human tumours and functional impact of mutations

The pooled distribution of mutated codons obtained after exposure to each of the three genotoxins discussed here allowed us to identify 6 major hot-spots in human tumours, codons 248, 273, 175, 245, 282, 249 (Figure 4).

At the phenotypic level, among the 63 mutants resulting from a nucleotide substitution, the two most frequently retrieved in human tumours were also found in our experimental patterns, i.e. Arg175His and Arg248Gln (Table 3). Moreover, four substitutions (Arg175His, Arg248Gln, Arg273Cys and Gly245Ser) represent together more than 10% of the mutants found in human tumours. Only three mutants found in our study have never been described in human tumours (Gly117Val, Gly262Leu, Asn288Ile). This strongly suggests that FASAY recovers mutants bearing a functional change allowing selection during the carcinogenesis process.

**Table 3:** Frequency in human tumours and functional impairment of some experimentally induced mutants.

Mutant	Aetiology(1)	Rank(2)	Nr of occurrence and frequency (%) in human tumours(3)	Loss of transcriptional activity(4)	Dominant negative effect(5)
Arg175His	AFB_1_ 17, B*[a]*P 12	1	1130 (4.25)	++	++
Arg248Gln	Ac 18–21, B*[a]*P 22–24	2	855 (3.21)	++	++
Arg273Cys	Ac 27–28	5	650 (2.44)	++	−
Gly245Ser	Ac 14–16, AFB_1_ 31–35	8	423 (1.60)	++	++
His179Tyr	B*[a]*P 14	19	104 (0.39)	++	++
Pro151Ser	AFB_1_ 12	27	96 (0.36)	++	+
Pro278Leu	Ac 29–30	37	79 (0.30)	++	+
Gly244Asp	AFB_1_ 27–28	56	60 (0.23)	++	++
Gly279Glu	B*[a]*P 31	69	48 (0.18)	++	++
Cys277Tyr	B*[a]*P 29	101	28 (0.11)	++	+
Arg267Trp	Ac 25–26	119	27 (0.10)	++	+
His179Asn	AFB_1_ 18–20	128	23 (0.09)	++	++
Arg283His	Ac 31–33, AFB_1_ 39, B*[a]*P 33–34	277	17 (0.06)	+	+/−
Ser127Pro	B*[a]*P 5	417	6 (0.02)	++	++

The table shows the 14 mutants induced by acetaldehyde (Ac), B*[a]*P or AFB_1_ for which exhaustive functional data were available from Dearth *et al.* [12].

(1) The number refers to the identity of the mutant in the corresponding paper: Ac, table 3 from [15]; AFB_1_, table 3 from [16]; B*[a]*P, table 2 from the present work.

(2) According to Dearth *et al.* [12].

(3) According to IARC database R14.

(4) ++: loss of transcriptional activity on the 22 p53 DBS tested; +: loss of transcriptional activity on 13 DBS only [12].

(5) ++: DNE on the 11 DBS tested; +: DNE on more than 5 DBS; +/−: DNE on 3 DBS only; −: no DNE [12].

We evaluated the functional consequence of mutations occurring after *in vitro* exposure to three chemicals in the light of the data from Dearth *et al.* that reviewed the impact of 76 *TP53* mutants frequently observed in human tumours [12]. Fourteen out of the 76 analysed mutants were retrieved in our three experimental patterns (Table 3). As expected, these 14 mutants were devoid of activity on *RGC* sequences which was used in our FASAY protocol. This analysis indicates that most of the experimental mutations harbour major functional impairment, i.e. complete loss of transcriptional activity on 22 p53 DNA Binding Sequences (DBS) and Dominant Negative Effect (DNE). Conversely, some of the mutations that are frequent in human tumours are unlikely to be retrieved by the FASAY because they retain transcriptional activity [12]. This includes for instance Arg175Leu or Glu285Lys that were not found in our assay.

However, the functional analysis of mutants by FASAY does not enable us to discriminate between different types of cancers. Indeed, if FASAY allow us to retrieve mutants occurring frequently in human tumours, these mutants were neither particularly frequent nor infrequent in tumours related to exposure to a particular genotoxin. For instance, Arg175His and Arg248Gln found in B*[a]*P and AFB_1_ patterns are the most frequently identified mutants in the human tumours, accounting for 4.25% and 3.21% of all mutants respectively (Table 3), but they account for only 1.2% of lung cancers or hepatocarcinoma mutants according to IARC database. In the same way, Arg273Cys depicted in the acetaldehyde pattern is more frequent in the whole human tumour spectrum (2.44%) than in that for oesophageal cancer (1.3%).

This analysis highlights the ability of chemicals to induce frequent and highly impaired *TP53* mutants which may be a key event involved in the carcinogenesis. Compared with classical mutagenesis assays using reporter genes, this functional *in vitro* approach provides strong arguments for a biological plausibility of carcinogenicity for a genotoxin. Nonetheless it recapitulates only the initiating step of carcinogenesis and consequently is inaccurate in predicting which particular mutation could be a driving force during the subsequent steps of carcinogenesis in a given tissue. This feature should be considered while comparing *in vitro* and *in vivo* patterns of mutants.

### Outlook

The FASAY performed after *in vitro* exposure of human normal fibroblasts to various genotoxins allowed us to depict experimental mutational patterns which share some, but not all, features of tumour patterns. This makes the FASAY a relevant and powerful tool to assess genotoxins implication in cancer aetiology, allowing a direct comparison of experimental and human cancer patterns of mutations. Indeed, demonstrating the ability of a chemical to induce experimentally the same deleterious mutations in a key tumour suppressor gene as found in relevant tumours provides a strong weight of evidence of human carcinogenicity. Our earlier data on acetaldehyde-induced mutations strongly suggest that alcohol acts as a true genotoxin in the development of head and neck and oesophageal tumours via production of acetaldehyde [15]. This supports the findings from epidemiological studies which have clearly shown that alcohol acts as a non-threshold carcinogen in such cancers [41]. The experimental AFB_1_-induced pattern [16] agreed with that found by other studies showing that G>T transversion in codon 249 found in hepatocarcinoma is likely a selection bias occurring during carcinogenesis [40], [42]. Finally the B*[a]*P pattern described in this study suggests that not only G>T transversions but also deletions could be the hallmark of B*[a]*P-exposure in lung tumours. Interestingly, a recent study using a massive parallel sequencing technology has revealed the mutational burden in a human small-cell lung cancer cell line, NCI-H209, after exposure to carcinogens in tobacco smoke [43]. The most prevalent mutations were G>T (34%), G>A (21%) and A>G (19%), which is in excellent agreement with mutations found in *TP53* in lung cancers. This highlights the fact that *TP53* mutational pattern can be considered as representative of the global mutational pattern arising during lung carcinogenesis, reinforcing the relevance of studying this gene as a key target for environmental carcinogens.

Finally, the ability of FASAY to study different biological materials including either normal and cancerous differentiated cells, or cancer stem cells, offers exciting perspectives to study mutational patterns in order to improve our knowledge of human chemical carcinogenesis.

## References

[1] Hainaut P, Hollstein M (2000). p53 and human cancer: the first ten thousand mutations.. Adv Cancer Res.

[2] Petitjean A, Mathe E, Kato S, Ishioka C, Tavtigian SV (2007). Impact of mutant p53 functional properties on TP53 mutation patterns and tumor phenotype: lessons from recent developments in the IARC TP53 database.. Hum Mutat.

[3] Luo JL, Tong WM, Yoon JH, Hergenhahn M, Koomagi R (2001). UV-induced DNA damage and mutations in Hupki (human p53 knock-in) mice recapitulate p53 hotspot alterations in sun-exposed human skin.. Cancer Res.

[4] Pfeifer GP, Denissenko MF, Olivier M, Tretyakova N, Hecht SS (2002). Tobacco smoke carcinogens, DNA damage and p53 mutations in smoking-associated cancers.. Oncogene.

[5] Smela ME, Currier SS, Bailey EA, Essigmann JM (2001). The chemistry and biology of aflatoxin B(1): from mutational spectrometry to carcinogenesis.. Carcinogenesis.

[6] Arlt VM, Stiborová M, vom Brocke J, Simões ML, Lord GM (2007). Aristolochic acid mutagenesis: molecular clues to the aetiology of Balkan endemic nephropathy-associated urothelial cancer.. Carcinogenesis.

[7] Besaratinia A, Pfeifer GP (2010). Applications of the human p53 knock-in (Hupki) mouse model for human carcinogen testing.. FASEB J.

[8] Bitton A, Neuman MD, Barnoya J, Glantz SA (2005). The p53 tumour suppressor gene and the tobacco industry: research, debate, and conflict of interest.. Lancet.

[9] Chen JX, Zheng Y, West M, Tang MS (1998). Carcinogens preferentially bind at methylated CpG in the p53 mutational hot spots.. Cancer Res.

[10] Tang MS, Zheng JB, Denissenko MF, Pfeifer GP, Zheng Y (1999). Use of UvrABC nuclease to quantify benzo[a]pyrene diol epoxide-DNA adduct formation at methylated versus unmethylated CpG sites in the p53 gene.. Carcinogenesis.

[11] Yoon J, Lee C, Pfeifer GP (2003). Simulated sunlight and benzo[a]pyrene diol epoxide induced mutagenesis in the human p53 gene evaluated by the yeast functional assay: lack of correspondence to tumor mutation spectra.. Carcinogenesis.

[12] Dearth LR, Qian H, Wang T, Baroni TE, Zeng J (2007). Inactive full-length p53 mutants lacking dominant wild-type p53 inhibition highlight loss of heterozygosity as an important aspect of p53 status in human cancers.. Carcinogenesis.

[13] Ishioka C, Frebourg T, Yan YX, Vidal M, Friend SH (1993). Screening patients for heterozygous p53 mutations using a functional assay in yeast.. Nat Gene.

[14] Flaman JM, Frebourg T, Moreau V, Charbonnier F, Martin C (1995). A simple p53 functional assay for screening cell lines, blood, and tumors.. Proc Natl Acad Sci U S A.

[15] Paget V, Lechevrel M, Sichel F (2008). Acetaldehyde-induced mutational pattern in the tumour suppressor gene TP53 analysed by use of a functional assay, the FASAY (functional analysis of separated alleles in yeast).. Mutat Res.

[16] Paget V, Sichel F, Garon D, Lechevrel M (2008). Aflatoxin B1-induced TP53 mutational pattern in normal human cells using the FASAY (Functional Analysis of Separated Alleles in Yeast).. Mutat Res.

[17] Scudiero DA, Shoemaker RH, Paull KD, Monks A, Tierney S (1988). Evaluation of a soluble tetrazolium/formazan assay for cell growth and drug sensitivity in culture using human and other tumor cell lines.. Cancer Res.

[18] Reddy MV, Randerath K (1986). Nuclease P1-mediated enhancement of sensitivity of 32P-postlabeling test for structurally diverse DNA adducts.. Carcinogenesis.

[19] Le Goff J, André V, Lebailly P, Pottier D, Périn F (2005). Seasonal variations of DNA-adduct patterns in open field farmers handling pesticides.. Mutat Res.

[20] Divi RL, Beland FA, Fu PP, Von Tungeln LS, Schoket B (2002). Highly sensitive chemiluminescence immunoassay for benzo[a]pyrene-DNA adducts: validation by comparison with other methods, and use in human biomonitoring.. Carcinogenesis.

[21] Shen YM, Troxel AB, Vedantam S, Penning TM, Field J (2006). Comparison of p53 mutations induced by PAH o-quinones with those caused by anti-benzo[a]pyrene diol epoxide in vitro: role of reactive oxygen and biological selection.. Chem Res Toxicol.

[22] Olivier M, Eeles R, Hollstein M, Khan MA, Harris CC (2002). The IARC TP53 database: new online mutation analysis and recommendations to users.. Hum Mutat.

[23] Cheng SC, Hilton BD, Roman JM, Dipple A (1989). DNA adducts from carcinogenic and noncarcinogenic enantiomers of benzo[a]pyrene dihydrodiol epoxide.. Chem Res Toxicol.

[24] Schurter EJ, Yeh HJ, Sayer JM, Lakshman MK, Yagi H (1995). NMR solution structure of a nonanucleotide duplex with a dG mismatch opposite a 10R adduct derived from trans addition of a deoxyadenosine N6-amino group to (−)-(7S,8R,9R,10S)-7,8-dihydroxy-9,10-epoxy-7,8,9,10-tetrahydrobenzo[a]pyrene.. Biochemistry.

[25] Yeh HJ, Sayer JM, Liu X, Altieri AS, Byrd RA (1995). NMR solution structure of a nonanucleotide duplex with a dG mismatch opposite a 10S adduct derived from trans addition of a deoxyadenosine N6-amino group to (+)-(7R,8S,9S,10R)-7,8-dihydroxy-9,10-epoxy-7,8,9,10- tetrahydrobenzo[a]pyrene: an unusual syn glycosidic torsion angle at the modified dA.. Biochemistry.

[26] Penning TM, Burczynski ME, Hung CF, McCoull KD, Palackal NT (1999). Dihydrodiol dehydrogenases and polycyclic aromatic hydrocarbon activation: generation of reactive and redox active o-quinones.. Chem Res Toxicol.

[27] Shou M, Harvey RG, Penning TM (1993). Reactivity of benzo[a]pyrene-7,8-dione with DNA. Evidence for the formation of deoxyguanosine adducts.. Carcinogenesis.

[28] Balu N, Padgett WT, Lambert GR, Swank AE, Richard AM (2004). Identification and characterization of novel stable deoxyguanosine and deoxyadenosine adducts of benzo[a]pyrene-7,8-quinone from reactions at physiological pH.. Chem Res Toxicol.

[29] Denissenko MF, Pao A, Tang M, Pfeifer GP (1996). Preferential formation of benzo[a]pyrene adducts at lung cancer mutational hotspots in P53.. Science.

[30] Smith LE, Denissenko MF, Bennett WP, Li H, Amin S (2000). Targeting of lung cancer mutational hotspots by polycyclic aromatic hydrocarbons.. J Natl Cancer Inst.

[31] Wei SJ, Chang RL, Bhachech N, Cui XX, Merkler KA (1993). Dose-dependent differences in the profile of mutations induced by (+)-7R,8S-dihydroxy-9S,10R-epoxy-7,8,9,10-tetrahydrobenzo(a)pyrene in the coding region of the hypoxanthine (guanine) phosphoribosyltransferase gene in Chinese hamster V-79 cells.. Cancer Res.

[32] Hakura A, Tsutsui Y, Sonoda J, Tsukidate K, Mikami T (2000). Comparison of the mutational spectra of the lacZ transgene in four organs of the MutaMouse treated with benzo[a]pyrene: target organ specificity.. Mutat Res.

[33] Yoon JH, Smith LE, Feng Z, Tang M, Lee CS (2001). Methylated CpG dinucleotides are the preferential targets for G-to-T transversion mutations induced by benzo[a]pyrene diol epoxide in mammalian cells: similarities with the p53 mutation spectrum in smoking-associated lung cancers.. Cancer Res.

[34] Aoki Y, Hashimoto AH, Amanuma K, Matsumoto M, Hiyoshi K (2007). Enhanced spontaneous and benzo(a)pyrene-induced mutations in the lung of Nrf2-deficient gpt delta mice.. Cancer Res.

[35] Xie Z, Braithwaite E, Guo D, Zhao B, Geacintov NE (2003). Mutagenesis of benzo[a]pyrene diol epoxide in yeast: requirement for DNA polymerase zeta and involvement of DNA polymerase eta.. Biochemistry.

[36] Liu Z, Muehlbauer KR, Schmeiser HH, Hergenhahn M, Belharazem D (2005). p53 mutations in benzo(a)pyrene-exposed human p53 knock-in murine fibroblasts correlate with p53 mutations in human lung tumors.. Cancer Res.

[37] Reinbold M, Luo JL, Nedelko T, Jerchow B, Murphy ME (2008). Common tumour p53 mutations in immortalized cells from Hupki mice heterozygous at codon 72.. Oncogene.

[38] Ruggeri B, DiRado M, Zhang SY, Bauer B, Goodrow T (1993). Benzo[a]pyrene-induced murine skin tumors exhibit frequent and characteristic G to T mutations in the p53 gene.. Proc Natl Acad Sci U S A.

[39] Culp SJ, Warbritton AR, Smith BA, Li EE, Beland FA (2000). DNA adduct measurements, cell proliferation and tumor mutation induction in relation to tumor formation in B6C3F1 mice fed coal tar or benzo[a]pyrene.. Carcinogenesis.

[40] Hussain SP, Schwank J, Staib F, Wang XW, Harris CC (2007). TP53 mutations and hepatocellular carcinoma: insights into the etiology and pathogenesis of liver cancer.. Oncogene.

[41] Baan R, Straif K, Grosse Y, Secretan B, El Ghissassi F (2007). WHO International Agency for Research on Cancer Monograph Working Group. Carcinogenicity of alcoholic beverages.. Lancet Oncol.

[42] Sohn S, Jaitovitch-Groisman I, Benlimame N, Galipeau J, Batist G (2000). Retroviral expression of the hepatitis B virus×gene promotes liver cell susceptibility to carcinogen-induced site specific mutagenesis.. Mutat Res.

[43] Pleasance ED, Stephens PJ, O'Meara S, McBride DJ, Meynert A (2010). A small-cell lung cancer genome with complex signatures of tobacco exposure.. Nature.

